# Does climate adaptation policy deliver a health dividend? Exposure, modeled health burden, and defensive capacity in China's climate-adaptive city pilots

**DOI:** 10.3389/fpubh.2026.1987084

**Published:** 2026-10-14

**Authors:** Cun Wang, Hantao Gong

**Affiliations:** School of Economics and Management, Nanchang Vocational University, Nanchang, China

**Keywords:** air pollution, climate adaptation policy, defensive capacity, digital infrastructure, modeled attributable mortality

## Abstract

Climate policy is widely expected to yield a health dividend: cleaner air can improve health without a simultaneous expansion of defensive capacity. We distinguish exposure reduction from defensive adaptation in China's 2017 climate-adaptive city pilot, using a 2011–2022 panel of 264 cities, including 21 treated units. A difference-in-differences design examines fine particulate matter (PM2.5) exposure, while the Global Exposure Mortality Model translates exposure into modeled pollution-related health burden. Physicians, hospital beds, and employee-insurance enrolment measure selected defensive inputs. The pilot is associated with a 3.87 μg/m^3^ lower PM2.5 (95% confidence interval −6.49 to −1.25; wild-bootstrap *P* = 0.0067). The estimate remains negative after pollution-convergence adjustments but is smaller and imprecise in the matched sample. The central accounting model implies an approximately 8.10% lower burden (conditional 95% confidence interval −11.32 to −4.76%), not an observed mortality reduction. Average capacity estimates are imprecise and cannot establish the absence of defensive adaptation. A physician-based coupling calibration admits positive and negative contributions and cannot bound the total defensive response. Among nine pre-policy capacity interactions, telecommunications intensity is positively associated with bed expansion (+4.40 per 10,000 residents; pointwise 95% confidence interval 2.47–6.32; adjusted *P* = 0.021), although pre-trend concerns limit causal interpretation. Other interactions remain uncertain. The main estimate is close to an illustrative detection threshold, warranting caution with non-significant findings. The results support an exposure-related health co-benefit under explicit accounting assumptions. Assessment incentives, coordination, and financing are possible explanations for uneven responses, but their roles are not identified by these estimates.

## 1 Introduction

Climate policy is routinely credited with a health dividend. Measures that reduce fossil energy use also reduce local air pollution, and reduced exposure to fine particulate matter (PM2.5) lowers premature mortality. This claim is central to the economic case for mitigation and has shaped a generation of policy appraisals; ambient particulate matter remains one of the largest environmental contributors to global mortality (1, 2), and quasi-experimental evidence from China documents its life-expectancy consequences (3, 4). A common empirical implementation follows one route: estimate the effect of a policy on emissions or ambient concentration, then apply an externally estimated concentration–response relationship to calculate pollution-related health benefits.

That route holds the concentration–response relationship fixed when translating reduced exposure into improved health. It can quantify an exposure-related health co-benefit under its assumptions, even if policy does not expand defensive capacity. It cannot, however, identify additional benefits from changes in the health system, early-warning infrastructure, or avoidance behavior that alter the consequences of a given concentration. If a policy moves both margins in the same direction, an exposure-only calculation may omit an additional defensive contribution. In this study, modeled PM2.5-attributable mortality is generated from pollution and fixed accounting inputs; it contains no observed deaths and cannot register defensive responses at a given exposure level.

The distinction matters because adaptation is not one technology but two. Deschênes and Greenstone (5) showed that the observed exposure–damage relationship is a net relationship that already contains the defensive response of households and firms; Barreca et al. (6) demonstrated that the relationship itself was rewritten over the 20th century as air conditioning diffused. On this reading, the health return to any environmental policy decomposes into an exposure channel and a defensive channel, and the two need not respond to policy at the same time or in the same direction. The broader climate-economics literature has made adaptation central for precisely this reason (7–10).

This study uses that decomposition to organize the empirical comparison. We define a coupling degree—the ratio of the defensive contribution to the exposure contribution—to compare their relative magnitudes. A positive coupling degree means the two channels reinforce each other and the health return exceeds what exposure alone explains. A coupling degree of zero means there is no net additional defensive contribution; a negative value means that contribution offsets part or all of the exposure gain. An exposure-related health co-benefit does not require positive coupling. Because the modeled burden contains no independent information on defensive adaptation, our physician-based calculation is a conditional illustration of one capacity component, not an estimate of total coupling.

Institutional design may influence the relative response of the two channels. Where a programme's assessment system rewards measurable, verifiable, and administratively attributable indicators, a multitask agency argument in the tradition of Holmström and Milgrom (11) suggests that effort may favor those tasks. Coordination between environmental and health administration and the timing of training and construction may also affect capacity responses. These considerations motivate possible explanations rather than determine the sign of coupling. The national pilot framework includes public health, early warning, emergency coordination, and supporting finance; it does not establish that the defensive margin was institutionally excluded.

We examine these margins in China's climate-adaptive city pilot, announced in 2017 for 28 localities, with 21 city-level treatment codes in our panel of 264 cities over 2011–2022. Some listed pilots cover districts, counties, or a cross-city area, so citywide coding represents a broader exposure proxy in four treated units. The programme explicitly concerns adaptation and permits both environmental and protective-service responses (12, 13). Because all pilots enter in a single 2017 cohort, comparisons between earlier- and later-treated cohorts do not arise; we nonetheless report the doubly robust estimator of Callaway and Sant'Anna (14) as a cross-check. With only 21 treated clusters, we report wild cluster bootstrap and permutation diagnostics alongside conventional clustered standard errors, while recognizing that designation was purposive.

Three findings follow. First, the pilot is associated with 3.87 μg/m^3^ lower PM2.5 exposure (95% confidence interval −6.49 to −1.25). The association remains negative under pollution-convergence and concurrent-policy controls, while the matched estimate is smaller and imprecise. The central Global Exposure Mortality Model (GEMM) calculation translates the exposure pattern into an approximately 8.10% lower modeled burden (conditional 95% confidence interval −11.32 to −4.76%). Second, average changes in physicians, beds, and employee-insurance enrolment are imprecisely estimated. These stocks do not reflect short-run protective services, and physician-based coupling intervals allow for a positive contribution. Third, the conversion-capacity interactions show a mixed pattern: higher pre-policy telecommunications intensity is associated with greater bed expansion, although pre-trend concerns limit causal interpretation. The remaining interactions are uncertain, and their non-significance does not indicate absent moderation.

The study contributes in three ways. Theoretically, it distinguishes exposure-related health accounting from the additional contribution of defensive adaptation and states what evidence would be needed to estimate their coupling. Empirically, it examines an adaptation programme through ambient PM2.5, its modeled health translation, and directly measured health-system inputs. This complements individual-level health evaluations of the same programme (15, 16), rather than supplying an independent mortality test. For policy, it clarifies the limits of drawing institutional conclusions from measured capacity. In a research area concerned with what artificial intelligence and digital infrastructure can do for low-carbon public health (17), our proxies measure infrastructure intensity and designation status rather than digital-governance quality. The results do not identify assessment criteria or cross-agency budget arrangements as binding constraints; testing those explanations requires local implementation and expenditure evidence.

## 2 Conceptual framework and hypotheses

### 2.1 Adaptation as two technologies

The theory organizing this article runs from a damage relationship to an incentive structure. Its anchor is the defensive-investment tradition in environmental and climate economics. Deschênes and Greenstone (5) established that the exposure–damage relationship recovered from observational data is a *net* relationship: households and firms respond to environmental stress by spending on protection, so the estimated slope already embeds that response. Barreca et al. (6) made this point historically, showing that the temperature–mortality relationship in the United States flattened over the 20th century as air conditioning diffused—the damage relationship itself was rewritten by defensive capacity. Graff Zivin and Neidell (18) and Neidell (19) document the behavioral counterpart: information about pollution changes avoidance, and avoidance changes the health consequences of a given concentration. Deschênes et al. (20) show that defensive expenditure is large enough to constitute a substantial share of the welfare gain from air quality regulation, and Moretti and Neidell (21) and Ito and Zhang (22) document the same margin in port and Chinese urban settings, respectively.

The implication is that adaptation is not one technology but two. Reducing exposure and raising defensive capacity are separate production processes. They may draw on different budgets, administrative competences, and time scales. Their contributions can be distinguished in a local decomposition of the health response, but their responsiveness to any particular policy is not guaranteed to be equal—or even to have the same sign. A programme may lower concentrations without expanding measured health-system stocks; another may improve protection through services or behavior at a given concentration.

A related account is the co-benefits framework, in which climate policy can improve health by lowering local pollutants alongside greenhouse gases (23–25). Its standard empirical implementation estimates the policy effect on emissions or concentrations and applies an externally estimated concentration–response relationship, an approach used in assessments of the health stakes of climate action (26, 27). That procedure holds the health-impact relationship fixed for the calculation. It need not assume an expansion of health-system capacity, and it remains informative about the exposure contribution. Our framework distinguishes that contribution from defensive responses that the calculation cannot observe.

### 2.2 The coupling between the two channels

Because the two technologies are separable, a local decomposition of the health return distinguishes what a policy achieves through each. It follows that the total return cannot be read off the exposure result alone: two policies that deliver identical air quality improvements may yield different health improvements if one also strengthens the defensive margin and the other does not.

We give this comparison a name. Let the coupling degree denote the size of the defensive contribution relative to the exposure contribution. It is a ratio, not a new parameter of the damage relationship, and it is informative because it is scale-free: it compares the additional defensive contribution with the contribution from exposure abatement. For a positive exposure contribution, three regimes follow.

When the coupling degree is positive, both channels push in the same direction, and the health return exceeds what exposure improvement alone accounts for. This reinforces defensive adaptation, but it is not a necessary condition for a health co-benefit. When the coupling degree is zero, the net defensive contribution is zero, and the health return equals the exposure contribution. When the coupling degree is negative, the defensive contribution works against the exposure gain and partially or fully offsets it, causing the return to fall short.

Two features of this formulation are important for what follows. First, it expresses the proposed mechanisms in comparable health units, rather than relying solely on sign patterns across outcomes. Second, locating the regime requires independent evidence on defensive responses and their health consequences. A mortality measure calculated from PM2.5 cannot distinguish between the regimes, as it contains only the exposure-based calculation. Physicians, beds, and enrolment provide additional evidence on selected administrative inputs, but do not account for warning systems, emergency services, preventive programmes, or household avoidance. An external physician elasticity therefore supports only a conditional calibration of that component; it cannot identify the full defensive contribution.

### 2.3 Institutional conditions and response timing

The framework is general; determining where the coupling degree lies is an empirical matter. Institutional design and response timing motivate three possible explanations for differences between exposure and defensive outcomes.

These considerations are hypotheses about implementation, not documented restrictions on the pilot's health activities. The 2016 work plan included public health, and the 2017 notification called for monitoring, early warning, emergency coordination, and supporting fiscal and financial policies (12, 13). Local scoring documents, budget records, and implementation reports are needed to establish how these provisions functioned in practice.

First, there is measurability asymmetry. Some environmental indicators can be measured frequently and attributed to administrative action more readily than service quality or prevention. In the multitask agency tradition of Holmström and Milgrom (11), when an agent allocates effort across tasks that differ in measurability, incentives attached to the measurable task may draw effort away from other tasks. The broader literature on local officials' promotion incentives highlights the importance of this possibility (28). However, applying it to this pilot requires evidence on local assessment weights; the present estimates do not establish that health-related activities were omitted from assessment.

Second, there is the issue of coordination across budgets and administrative responsibilities. Environmental improvements and health protection can involve different departments, financing arrangements, and personnel decisions. Such boundaries may affect the timing and composition of local action, but the national framework also anticipates interdepartmental coordination and financial support. The presence of separate responsibilities does not establish that municipalities lacked the authority to invest in health protection.

Third, there is a time-scale mismatch. Physician training and hospital construction may adjust more slowly than exposure or service delivery. Within an evaluation window, investment may therefore be incompletely reflected in capacity stocks. Early warnings, risk messaging, emergency coordination, and preventive services can change over shorter horizons, so an imprecise stock response does not rule out defensive adaptation.

Together, these considerations motivate the examination of differences in the timing and scale of responses. They do not determine the sign of coupling: a small or imprecisely estimated stock response is compatible with a positive contribution through unobserved protective services.

### 2.4 Conversion capacity as a boundary condition

The relevance of these considerations may vary across cities. Monitoring costs, cross-agency coordination, and fiscal resources all vary, and each may influence implementation. Where information infrastructure is dense, monitoring, early warning, and exposure-avoidance communication may be easier. Integrated information systems may facilitate coordination, and fiscal resources may support services that do not require long construction cycles. Telecommunications intensity, smart-city status, and expenditure intensity are imperfect proxies for these conditions: they do not directly measure the quality or reach of protective services.

Figure 1 summarizes the framework: the two channels, possible influences on implementation, and the point at which conversion capacity may intervene. We call the resulting city-level attribute conversion capacity: the ability to turn a policy requirement into effective action. If conversion capacity supports exposure reduction or defensive services, cities holding more of it may show larger improvements. A weak interaction, however, cannot by itself locate an institutional ceiling because proxy quality, limited precision, and response timing also affect the estimates.

**Figure 1:**
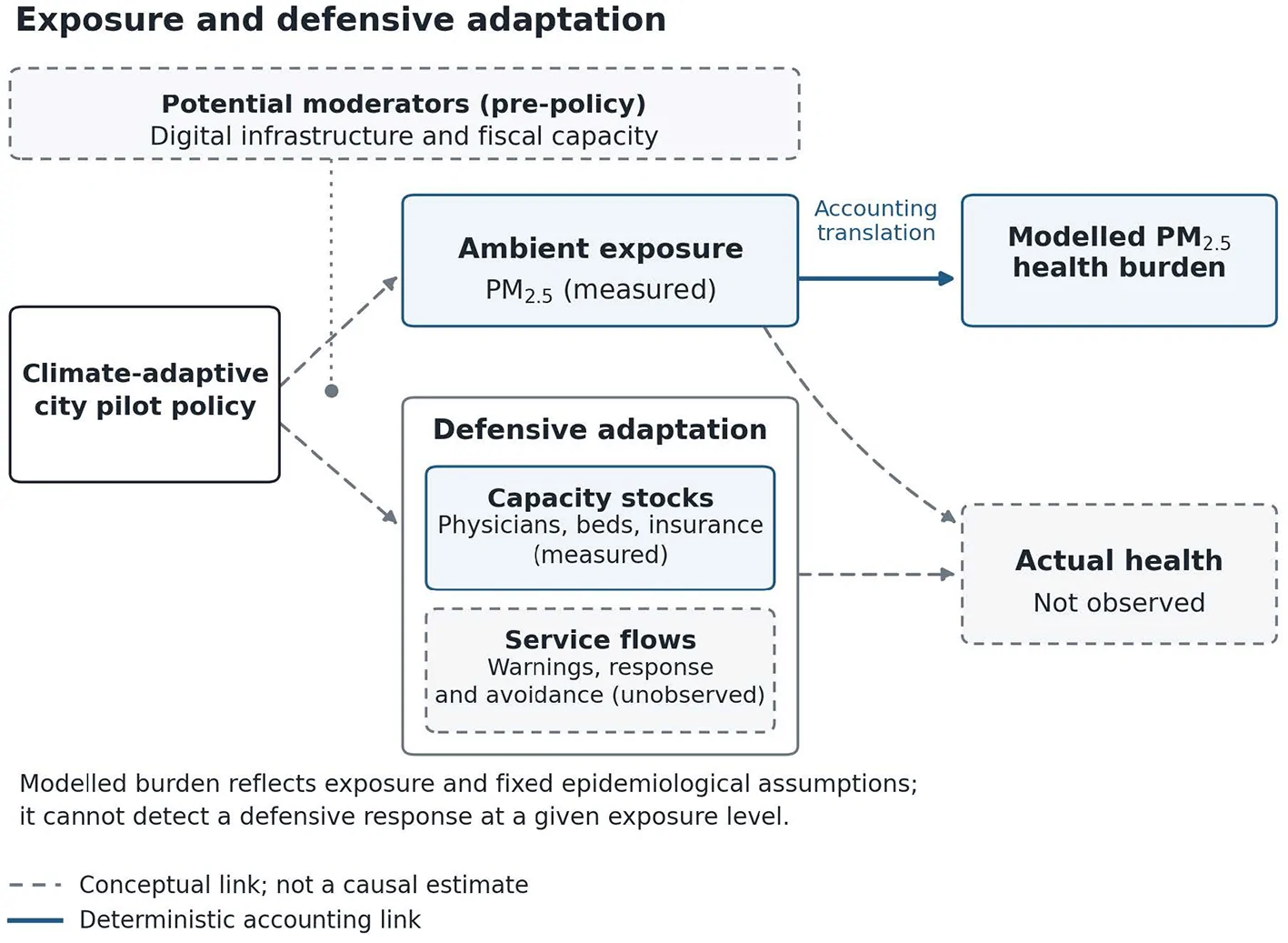
Theoretical framework. The climate-adaptive city pilot may affect exposure and defensive adaptation through distinct pathways. Modeled PM2.5-attributable mortality is calculated from exposure and fixed accounting inputs, while actual health is not observed. Physicians, beds, and enrolment capture selected defensive inputs; protective-service flows remain unobserved. Arrows denote conceptual links rather than estimated causal effects, with the exposure-to-modeled-burden link representing the accounting calculation. Baseline digital and fiscal inputs are potential moderators.

### 2.5 Hypotheses

The pilot's urban planning, infrastructure, and environmental management provisions could reduce pollution exposure. Under a monotone concentration–response relationship and fixed accounting inputs, such a reduction would also lower the modeled pollution-related burden. This motivates the directional hypothesis, whose direct empirical implication is lower PM2.5.

H1a (direction). The climate-adaptive city pilot is associated with a reduction in modeled PM2.5-attributable mortality.

H1b (magnitude). The defensive contribution does not add to the exposure contribution within the evaluation window, so the coupling degree is non-positive.

H1b is a restriction on the additional defensive contribution, not a distinction between our framework and the co-benefits account. Exposure reduction alone can yield a health co-benefit. Establishing H1b would require sufficiently informative evidence on defensive responses and their health effects. Failure to detect a stock increase, or a physician-based interval that includes positive values, does not establish the restriction.

H2 (conversion capacity). The association between the pilot and lower modeled pollution-related burden is stronger where pre-policy conversion capacity is greater, measured by (a) telecommunications intensity, (b) smart-city pilot status, and (c) fiscal expenditure intensity.

The three carriers are of different provenance—digital, governance, and fiscal—and are read as a group because it is the construct rather than any single indicator that the hypothesis concerns. For the modeled burden, H2 concerns the moderation of an exposure-based outcome. A defensive interpretation additionally requires evidence on directly measured capacity or services, so we examine capacity outcomes separately. The interactions are interpreted jointly within each test family, with their uncertainty taken into account. Non-significant interactions do not establish equivalence or imply that assessment criteria and budget boundaries are the binding constraints; positive interactions with administrative stocks do not by themselves establish improved health.

## 3 Materials and methods

### 3.1 Sample and data

The sample is a panel of 264 Chinese prefecture-level cities observed annually from 2011 to 2022 (3,152 city-years; 3,135 from 262 cities enter the main regression after listwise deletion on the estimating variables). The available exposure, registered population, and covariate series jointly determine the observation window. The window contains six pre-policy years (2011–2016) and six years from designation onward (2017–2022). The national announcement names 28 localities at different administrative levels; 21 panel cities are coded as exposed. Chongqing, Xi'an, Xianyang, and Xining represent broader areas than the named districts, county, or cross-city new area. Appendix Table A7 documents these mappings, and a sensitivity analysis excludes the four broader-area proxies.

Four sources are merged on city administrative code and year. City economic, fiscal, education, health, and telecommunications variables are drawn from the China City Statistical Yearbook and the China Urban Construction Statistical Yearbook. Ambient PM2.5, industrial sulfur dioxide emissions, and the share of days meeting air quality standards come from a prefecture-level environmental regulation and pollution database covering 2000–2024, with PM2.5 retrieved from satellites. Climate variables are aggregated to the city-year from NASA POWER daily reanalysis at city centroids. Pilot lists for the climate-adaptive city programme and for the concurrent policies used as controls are hand-coded from published ministerial documents.

Continuous unbounded regression variables are winsorised at the 1st and 99th percentiles during panel construction. Binary indicators, policy difference-in-differences terms, and bounded shares are not winsorised. Health-accounting variables are derived from processed PM2.5 and registered population. Listwise deletion is applied to the variables entering each specification; additional fixed effects may remove singleton observations. Consequently, estimation samples need not coincide across baseline columns or outcomes, and observation counts are reported separately.

### 3.2 Variables and measurement

The exposure and defensive constructs in Section 2 map to separate city-year indicators. Annual PM2.5 concentration is the primary observed outcome. The modeled health burden is a secondary accounting quantity whose interpretation depends on the concentration–response function and fixed demographic assumptions.

#### 3.2.1 Outcomes: PM2.5 exposure and modeled attributable mortality

Annual mean satellite-derived PM2.5, measured in μg/m^3^, records city-level ambient exposure rather than individual inhaled dose. The study does not contain a city-year panel of observed cause-specific deaths suitable for the evaluation window. The health burden is therefore an accounting quantity computed from exposure, not an observed count of deaths. It cannot detect changes in medical care, warnings, or household protection at a given concentration because these responses do not enter its formula.

The construction follows the Global Exposure Mortality Model (GEMM) of Burnett et al. (29), using the pooled non-communicable-disease-plus-lower-respiratory-infection hazard function for ages 25 and above. For city *i* in year *t* with an annual mean PM2.5 concentration (μg/m^3^), let *z*_*it*_ = max(0, *c*_*it*_ − *c*_0_) be concentration above the counterfactual floor. The hazard ratio is defined in Equation 1 as


$$\begin{array}{l} HR\left(c_{it}\right)=\exp\left\{\frac{\theta \ln\left(1+\frac{z_{it}}{\alpha }\right)}{1+\exp\left(-\frac{\left(z_{it}-\mu \right)}{\nu }\right)}\right\}, \end{array}$$ (1)

with GEMM parameters θ = 0.1430, α = 1.6, μ = 15.5, ν = 36.8, and *c*_0_ = 2.4 μg/m^3^. The attributable fraction is calculated using Equation 2 as


$$\begin{array}{l} AF_{it}=\frac{HR\left(c_{it}\right)-1}{HR\left(c_{it}\right)}. \end{array}$$ (2)

The modeled attributable death rate, per 100,000 adults aged 25 and above is calculated using Equation 3 by applying the attributable fraction to an assumed common baseline mortality rate B:


$$\begin{array}{l} R_{it}=AF_{it}\times B\times k, \end{array}$$ (3)

Here, B = 868 per 100,000 adults is a fixed accounting assumption, approximately consistent with a crude mortality rate of 7.1 per thousand, an 88% share of the included causes, and an adult share of 0.72. The constant k = 0.4477 is a common normalization. These are not city-specific mortality measurements, and the normalization does not validate absolute local death counts. Multiplicative constants are absorbed exactly by an intercept when the outcome is ln(M) or ln(R); this invariance is only approximate for ln(1 + M). A separate log-rate specification removes population weighting.

Modeled attributable deaths are obtained by applying the modeled attributable death rate to the assumed exposed population, as shown in Equation 4:


$$\begin{array}{l} M_{it}=\frac{R_{it}}{10^{5}}\times \left(0.72\times Pop_{it}\right), \end{array}$$ (4)

Here, Pop is the registered population in persons and 0.72 is the assumed population share aged 25 and above. The secondary dependent variable is defined in Equation 5 as follows:


$$\begin{array}{l} Y_{it}=ln\left(1+M_{it}\right). \end{array}$$ (5)

Appendix Figure A1 sets out this accounting chain and plots the attributable fraction over the observed concentration range. The rate is a monotonic function of PM2.5; the count additionally weights it by population. Neither includes an independent mortality observation or information on defensive adaptation. PM2.5 and its health translation therefore enter separate outcome regressions, not opposite sides of a regression interpreted as a health mechanism. Appendix Table A2 varies the published GEMM risk parameter and uses the alternative fit excluding the Chinese male cohort; additional floor changes are stress tests. Each policy interval is conditional on its parameter scenario and does not combine regression uncertainty with all epidemiological, exposure, and demographic uncertainty.

#### 3.2.2 Core regressor: the climate-adaptive city pilot

The treatment is the difference-in-differences term defined in Equation 6:


$$\begin{array}{l} {\text{Pilot}}_{it}={\text{Treat}}_{i}\times 𝟙\left(t\geq 2017\right), \end{array}$$ (6)

Treat equals one for the 21 panel cities coded as exposed to the 2017 climate-adaptive city pilot. Some codes represent parent-city or cross-boundary exposure proxies rather than whole-city designations, as described in Section 3.1. All pilots enter in a single cohort, avoiding comparisons between earlier- and later-treated cohorts that can complicate staggered adoption (30, 31). The specification uses the contemporaneous treatment indicator. The common start date does not remove concerns about purposive selection, differential trends, or spillovers.

#### 3.2.3 Moderators: three carriers of conversion capacity

##### 3.2.3.1 Digital carrier

Telecommunications intensity is ln(1 + telecommunications revenue per resident), with revenue and population expressed in compatible units. It proxies information infrastructure rather than the reach or quality of implemented digital governance. The primary moderation analysis fixes the measure at its 2016 value, then centers and scales it by the cross-city standard deviation.

##### 3.2.3.2 Governance carrier

Smart-city pilot status is a 0/1 indicator of whether the city had entered the separate national smart-city pilot programme by 2016. The programme concerns urban information infrastructure and has been studied in relation to energy and emissions (32), but designation alone does not measure the quality of cross-departmental coordination. The indicator retains its binary scale.

##### 3.2.3.3 Fiscal carrier

Fiscal expenditure intensity is general public budget expenditure divided by gross regional product. Its 2016 value proxies pre-policy fiscal inputs and is standardized across cities. The baseline regression separately controls for contemporaneous fiscal intensity. Fixing the moderator before the adaptation pilot avoids treating a potentially policy-induced fiscal change as a predetermined capacity. Comparisons with time-varying moderators appear in Appendix Table A5.

#### 3.2.4 Channel outcomes

##### 3.2.4.1 Exposure margin

(i) PM2.5 concentration, satellite-derived annual mean, μg/m^3^. (ii) ln(1 + industrial sulfur dioxide emissions). (iii) Share of days meeting air quality standards, percentage points. PM2.5 is the primary outcome; the other two provide supplementary environmental comparisons. The design does not establish that each measure was a formal assessment target of the adaptation programme.

##### 3.2.4.2 Defensive margin

(i) Physicians per 10,000 registered residents. (ii) Hospital and health center beds per 10,000 registered residents. (iii) Employee basic medical insurance enrolment divided by registered population, estimated for 2016–2022 because the earlier enrolment series is not consistently defined. This ratio is not universal insurance coverage and has only one pre-policy year. Physician supply is associated with population mortality in other settings (33, 34), but these administrative stocks do not measure warning reach, emergency response performance, prevention, service quality, or household avoidance. Confidence intervals are reported in natural units and relative to sample means; non-significance alone does not establish the absence of defensive adaptation.

#### 3.2.5 Controls

The control vector absorbs development level, demographic scale, fiscal capacity, industrial structure and climatic background: log gross domestic product (GDP) per capita; log registered population; fiscal expenditure over gross regional product (GRP); secondary and tertiary value-added shares (percentage points); annual mean temperature (°C); ln(1 + annual precipitation); and days with maximum temperature at or above 35 °C. Because economic and fiscal controls could themselves respond to policy, a climate-controls-only specification assesses dependence on these potentially intermediate variables.

Table 1 lists the variables and descriptive statistics. The source families are described in Section 3.1, and the replication data dictionary records definitions, units, and availability.

**Table 1:** Variable definitions and descriptive statistics.

Variable	Role	Measurement	*N*	Mean	SD
Log modeled deaths	Accounting outcome	ln(1 + modeled deaths)	3,152	7.881	0.814
Modeled deaths	Accounting level	Modeled persons per city-year	3,152	3,532.668	2,788.270
Modeled rate	Accounting variant	Per 100,000 adults aged 25+	3,152	101.118	22.594
Pilot	Treatment	Pilot city × 1(year ≥ 2017)	3,152	0.040	0.196
Ln telecom p.c.	Digital proxy	ln(1 + telecom revenue/population)	3,148	6.673	0.704
Smart-city pilot	Governance proxy	0/1 from smart-city entry	3,152	0.509	0.500
Fiscal expenditure/GRP	Fiscal proxy; control	Budget expenditure/GRP	3,152	0.195	0.093
PM2.5	Primary exposure	Satellite annual mean, μg/m^3^	3,152	42.806	16.181
Ln industrial SO_2_	Exposure comparison	ln(1 + industrial SO_2_)	3,152	1.107	0.752
Good-air-day share	Exposure comparison	% of days meeting standard	3,152	79.807	15.567
Physicians per 10k	Defensive stock	Physicians per 10,000 residents	3,152	25.305	11.915
Beds per 10k	Defensive stock	Beds per 10,000 residents	3,152	47.841	17.273
Employee-insurance ratio	Defensive enrolment	Employee enrolls/population	1,844	0.227	0.203
Ln GDP per capita	Control	ln(GRP per capita)	3,152	10.822	0.554
Ln population	Control	ln(registered population)	3,152	5.921	0.691
Secondary share	Control	Secondary value added/GRP, %	3,152	45.459	10.690
Tertiary share	Control	Tertiary value added/GRP, %	3,152	43.233	10.152
Mean temperature	Control	Annual mean, °C	3,135	14.625	5.050
Ln precipitation	Control	ln(1 + annual precipitation)	3,135	6.853	0.581
Heat days	Control	Days with maximum ≥35 °C	3,135	9.805	13.821

Descriptive statistics use each variable's available observations before regression-specific restrictions. The three moderator rows describe the source series; primary interactions fix the 2016 value and standardize continuous proxies across cities. Insurance is available over 2016–2022. Modeled mortality quantities are accounting outputs, not observed deaths.

SD, standard deviation.

### 3.3 Empirical strategy

#### 3.3.1 Baseline

The primary exposure association and the modeled burden implication of H1a are estimated separately by two-way fixed effects,


$$\begin{array}{l} Q_{it}=\beta {\text{Pilot}}_{it}+X_{it}^{\prime }\gamma +\mu_{i}+\lambda_{t}+\varepsilon_{it}, \end{array}$$ (7)

Here Q denotes the outcome, with city effects μ, year effects λ, and standard errors clustered by city. H1a predicts a negative modeled-burden coefficient; its direct exposure implication is lower PM2.5. Table 2 presents the PM2.5 specification with city and year effects, then adds climate controls and the full control vector. The final column replaces year effects with province-by-year effects to absorb regional shocks and policy timing. Causal interpretation requires conditional parallel trends, no differential anticipation, and no relevant spillovers or unobserved concurrent shocks.

**Table 2:** The climate-adaptive city pilot and PM2.5 exposure.

Specification	Estimate	95% CI	*P-*value	*N*	Cities/pilots
City/year effects	−4.18	−7.09, −1.28	0.005	3,152	264/21
Climate controls	−3.98	−6.85, −1.11	0.007	3,135	262/21
Full controls	−3.87	−6.49, −1.25	0.004	3,135	262/21
Province/year effects	−3.83	−5.80, −1.87	< 0.001	3,078	258/20

Dependent variable: annual PM2.5 concentration, μg/m^3^. All specifications contain city fixed effects. The first three use year effects; the fourth uses province-by-year effects. Climate controls are temperature, precipitation, and heat days; full controls add the socioeconomic variables in Section 3.2.5. Confidence intervals and P-values use city-clustered inference. Observation, city, and pilot counts vary with covariate availability and singleton removal. PM2.5 is the primary outcome; the modeled burden translation is reported separately.

#### 3.3.2 Dynamics

The event study replaces the treatment term in Equation 7 with treated-city indicators for event time relative to 2017. The omitted reference is 2016; 2011–2012 are pooled at 5 or more years before treatment, and 2022 is event year five. Joint pre-period tests use clustered and wild-bootstrap inference. Non-rejection is a diagnostic with limited power rather than proof of parallel trends.

#### 3.3.3 Channels

Equation 7 is re-estimated for each exposure and defensive outcome, retaining the treatment coding, full control vector, city/year effects, and city clustering. Outcome-specific availability determines the sample; insurance is observed only from 2016. The capacity regressions examine measured inputs rather than the entire defensive contribution.

#### 3.3.4 Moderation

Conversion capacity enters through a fixed pre-policy interaction, as specified in Equation 8:


$$\begin{array}{c} Q_{it}=\beta {\text{Pilot}}_{it}+\delta \left({\text{Pilot}}_{it}C_{i}\right)+X_{it}^{\prime }\gamma +\mu_{i}+\lambda_{t}\\ +\underset{t}{\sum }\rho_{t}C_{i}1\left(\text{year}=t\right)+u_{it}. \end{array}$$ (8)

Here C is the 2016 carrier, standardized for continuous measures and binary for smart-city status. Its level is absorbed by city effects, and carrier-by-year terms permit different common time paths by baseline capacity. H2 predicts a negative interaction for modeled burden. Direct capacity interactions are exploratory extensions testing whether the same proxies predict larger changes in measured defensive inputs.

#### 3.3.5 Identification and inference

Identification rests on within-city variation net of annual shocks. With 21 treated cities, conventional cluster inference may be unreliable (35, 36), so we also report null-imposed wild cluster bootstrap tests with 9,999 Rademacher draws. A permutation diagnostic uses 1,999 reassignments of 21 pilot cities while retaining the 2017 date and reports coefficient-based and studentised *P*-values. Because designation was purposive, this is not an exact randomization test for the historical assignment. Estimator comparisons use doubly robust difference-in-differences (14), entropy balancing on 2016 covariates (37), and 1:3 propensity-score matching. Trend checks interact pre-policy pollution with years, including a 2011–2012 baseline preceding the 2013 national air pollution action plan. Holm (38) correction is applied separately to the three exposure tests, six exposure/burden interactions, and nine direct-capacity interactions. Intervals remain pointwise rather than simultaneous. A false policy date of 2014 is evaluated within 2011–2016.

## 4 Results

### 4.1 Descriptive statistics

Table 1 reports the available city-year observations before outcome-specific estimation restrictions. Ambient PM2.5 averages 42.81 μg/m^3^, with a standard deviation of 16.18. In the complete-case benchmark, its mean is 42.87 across 3,135 observations from 262 cities, including all 21 treated cities. Physicians and hospital beds average 25.30 and 47.84 per 10,000 residents in the full panel. Appendix Figure A1 displays raw group trends; differences in these means do not themselves identify the policy association. Modeled burden is reported separately because its variation is derived from PM2.5, population, and fixed accounting assumptions.

### 4.2 Baseline: the pilot and PM2.5 exposure

H1a predicts a reduction in modeled PM2.5-attributable mortality. Table 2 first tests its direct exposure implication before applying the health-impact model. With city and year effects, the PM2.5 coefficient is −4.18 μg/m^3^; adding climate controls gives −3.98. The full-control estimate is −3.87 (95% confidence interval −6.49 to −1.25), about 9.0% of the estimation sample mean. Province-by-year effects yield −3.83 (−5.80 to −1.87). The negative association is thus present without the contemporaneous socioeconomic controls, although interpretation still depends on the counterfactual trend assumptions.

Applying the central GEMM formula to the exposure and population series gives a coefficient of −0.0844 for ln(1 + modeled attributable deaths), with a 95% confidence interval of −0.1201 to −0.0487. This implies an approximately 8.10% lower modeled burden (4.76%−11.32% lower, conditional on the model). The log attributable-rate coefficient is −0.0847. These estimates describe an exposure-based health impact translation, not observed deaths or an independent mortality test. Model sensitivity appears in Section 4.5 and Table 4A; the modeled burden event study is Appendix Figure A5.

### 4.3 Dynamics and identification

Figure 2 plots the PM2.5 event study. Pre-policy coefficients are jointly indistinguishable from zero under conventional clustered inference (*P* = 0.717) and the wild bootstrap (*P* = 0.776). The post-policy coefficients are negative throughout the observation window. The timing is consistent with the pilot comparison, but the pre-period intervals also permit deviations from common trends. These diagnostics do not establish the identifying assumption.

**Figure 2:**
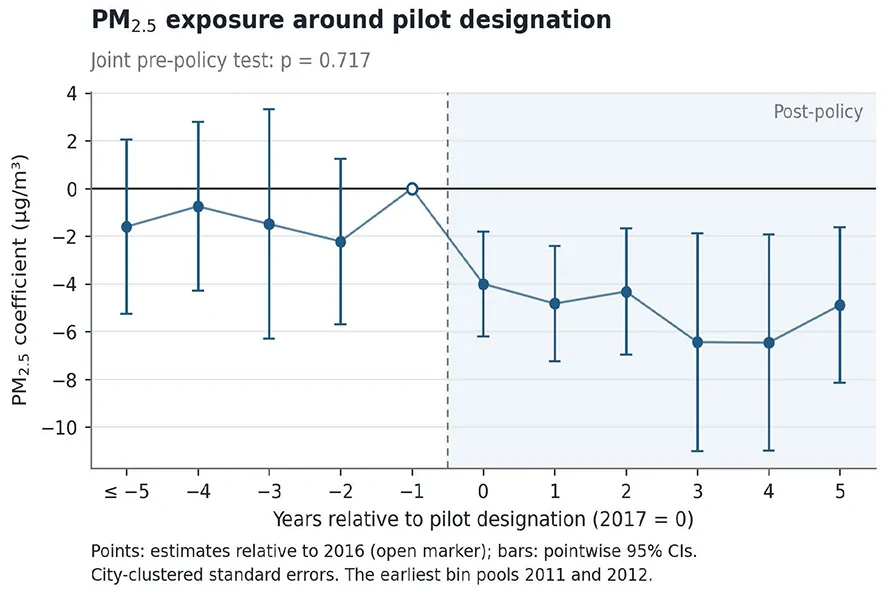
PM2.5 event study. Coefficients and pointwise 95% city-clustered confidence intervals are relative to 2016; years 2011–2012 are pooled. The vertical line marks the 2017 policy start. The pre-period joint *P*-values are 0.717 conventionally and 0.776 under the wild bootstrap.

Estimator choice changes magnitude and precision (Appendix Table A1). The doubly robust PM2.5 estimate is −3.94 μg/m^3^ (95% confidence interval −6.32 to −1.56), and entropy balancing yields −2.98 (−5.59 to −0.37). The matched-panel estimate is smaller and statistically imprecise: −1.67 (−4.92 to +1.57; *P* = 0.308; *N* = 874). The association is therefore not established at 5% under every estimator. Matching changes both the comparison sample and the magnitude, while the modeled burden comparisons remain conditional on the accounting function.

### 4.4 Channel attribution: exposure and measured defensive capacity

Table 3 compares the exposure and defensive margins using a common specification and outcome-specific samples. PM2.5 is the primary exposure outcome. The auxiliary coefficient is −0.211 log points for ln(1 + industrial sulfur dioxide emissions), while the good-air-day share rises by 4.164 percentage points. Holm correction of the three wild-bootstrap *P*-values gives 0.020 for PM2.5, 0.042 for sulfur dioxide, and 0.055 for good-air days; the last is not established at 5%. These environmental comparisons concern exposure and emissions, not the health consequences of additional defensive services.

**Table 3:** Exposure outcomes and measured defensive capacity.

Outcome	Estimate	95% CI	Wild *P*	Holm *P*	*N*
PM2.5	−3.874	−6.494, −1.253	0.007	0.020	3,135
Ln industrial SO_2_	−0.2113	−0.3727, −0.0499	0.021	0.042	3,135
Good-air days	4.164	0.196, 8.132	0.055	0.055	3,135
Physicians/10k	0.284	−1.335, 1.903	0.736	—	3,135
Beds/10k	−0.617	−3.375, 2.140	0.667	—	3,135
Employee insurance	0.0041	−0.0105, 0.0187	0.584	—	1,832

All rows use the full control vector, city/year fixed effects, and city clustering. The outcome determines the estimation sample; insurance covers 2016–2022 only. Intervals are pointwise 95% city-clustered intervals. Wild-bootstrap P-values use 9,999 null-imposed draws. The Holm correction applies to the three-exposure P-values as one family; the three average-capacity P-values are shown unadjusted. Neither non-significance nor the plotted interval establishes equivalence to zero.

On the defensive margin, the number of physicians per 10,000 residents changes by +0.284 (95% confidence interval −1.335 to +1.903), or +1.12% of their estimation-sample mean (−5.29% to +7.53%). The number of beds changes by −0.617 (−3.375 to +2.140), or −1.29% (−7.06% to +4.48%). The corresponding wild-bootstrap ranges are −5.72% to +7.88% and −7.55% to +4.88% of the means. The employee-insurance ratio changes by +0.0041 (−0.0105 to +0.0187) over 2016–2022. These estimates are imprecise, and no equivalence threshold was specified. Bed-stock pre-policy tests reject joint zero (conventional *P* = 0.0087; wild *P* = 0.0231), further limiting causal interpretation.

H1b predicts that the defensive contribution is non-positive relative to the exposure contribution. The modeled burden cannot test that restriction because it contains no defensive input. Physician supply offers an illustrative calibration of one measured component, conditional on an external physician–mortality elasticity. It cannot determine the sign of overall coupling or bound unobserved protective services and behavior.

Basu et al. (33) report an association between primary-care physician supply and mortality in the United States. That study motivates an illustrative elasticity of −0.047, which is not an identified Chinese parameter: its physician definition, cause mix, population, and health system differ from this setting. The calibration scales the proportional physician-density coefficient by the assumed elasticity and divides it by the negative log modeled-rate coefficient. At −0.047, the ratio is 0.0062, with a conditional 95% Fieller interval of (−0.0324, +0.0462). Formally,


$$\begin{array}{c} \kappa_{D}=\frac{-\eta_{D}\left(\Delta D/\bar{D}\right)}{-\beta_{R}}. \end{array}$$ (9)

In Equation 9, ΔD is the physician-density coefficient, the denominator of its proportional change is the estimation-sample mean physician density, ηD is the assumed physician–mortality elasticity, and βR is the pilot coefficient for the log modeled attributable rate. The ratio concerns the physician component only.

The Fieller interval propagates the joint city-clustered regression uncertainty in the physician and exposure-accounting coefficients while holding the external elasticity and sample-mean scaling fixed. At an elasticity of −0.10, the ratio is 0.0133 (95% interval −0.0690 to +0.0982); at −0.50, it is 0.0663 (−0.3449 to +0.4912). Appendix Table A3 provides the full grid and separate fixed-denominator bootstrap ranges. These are assumption-sensitive physician scenarios, not total defensive bounds. Positive contributions remain compatible with the evidence, so H1b is not established. Figure 3 compares the observed outcome coefficients on a standardized scale.

**Figure 3:**
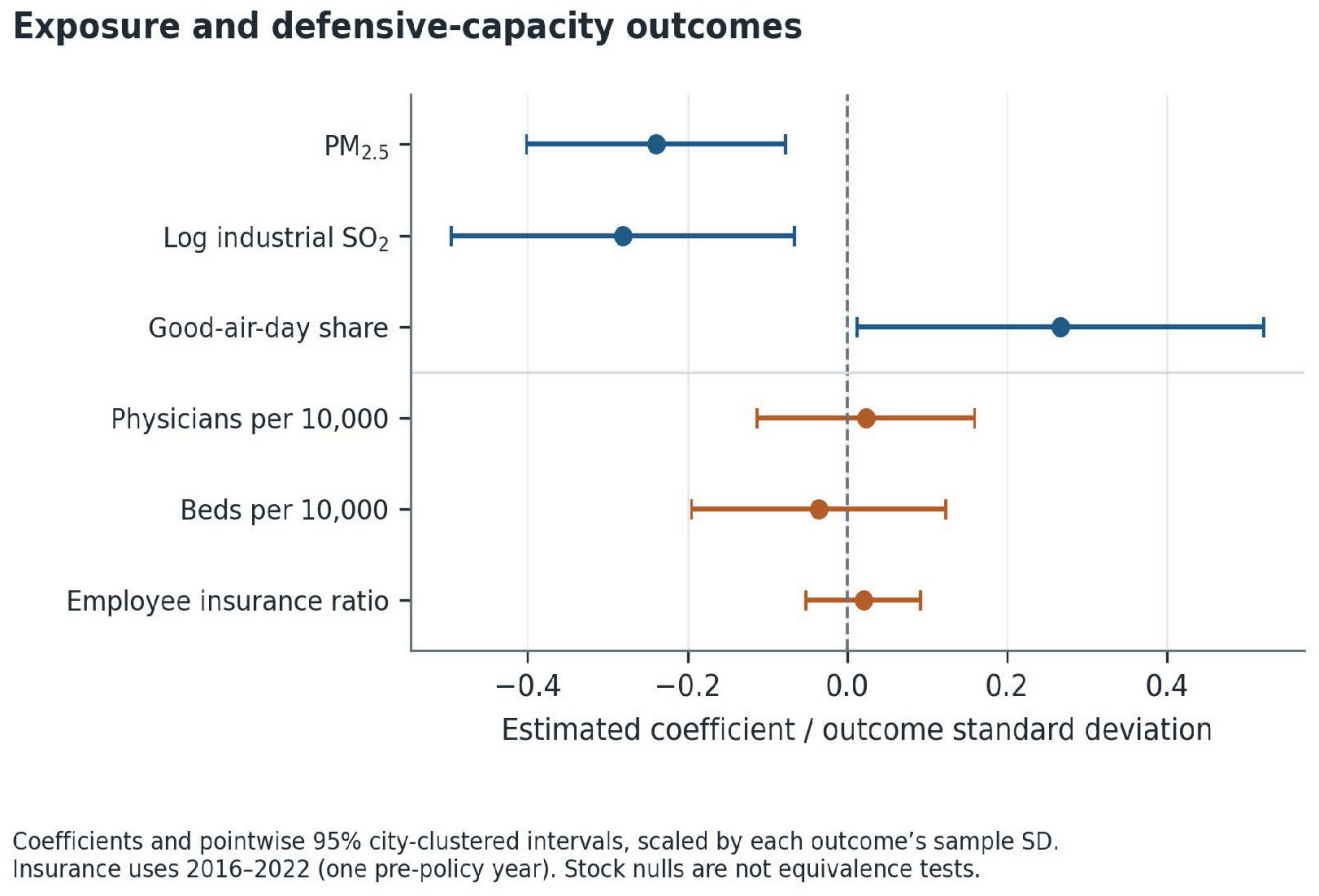
Exposure and defensive outcomes on a common standardized scale. Coefficients and pointwise 95% city-clustered intervals are divided by each outcome's estimation-sample standard deviation. These administrative stocks and enrolment do not measure warning systems, emergency response, preventive services, or household avoidance.

### 4.5 Robustness and inference

Table 4 reports PM2.5 sensitivity to trends, concurrent policies, and spatial coding. The coefficient is −3.65 μg/m^3^ with five concurrent policy controls, −3.77 after excluding municipalities, and −3.61 after omitting 2020. Excluding Chongqing, Xi'an, Xianyang, and Xining, whose citywide treatment codes represent broader areas than the named pilot localities, yields −4.32 (95% confidence interval −7.29 to −1.36) with 17 treated cities. These checks reduce specific confounding and coding concerns without establishing that all concurrent influences have been removed.

**Table 4:** PM2.5 robustness to trends, policies, and spatial coding.

Specification	Estimate	95% CI	*P-*Value	*N*	Pilots
2016 PM2.5 × year	−3.21	−5.65, −0.77	0.010	3,130	21
2016 PM2.5 tercile × year	−3.70	−5.89, −1.50	0.001	3,130	21
2011–2012 PM2.5 × year	−3.64	−5.49, −1.79	< 0.001	3,120	21
Five concurrent policies	−3.65	−6.17, −1.12	0.005	3,135	21
Exclude 2020	−3.61	−6.35, −0.86	0.010	2,873	21
Exclude municipalities	−3.77	−6.47, −1.07	0.006	3,087	20
Exclude partial-area mappings	−4.32	−7.29, −1.36	0.004	3,087	17

All specifications contain full controls and city/year effects. Intervals are pointwise 95% city-clustered intervals. The concurrent controls are the separate low-carbon city, carbon-trading, zero-waste city, green-credit, and environmental-court policies. Partial-area exclusions are Chongqing, Xi'an, Xianyang, and Xining. The wild-bootstrap benchmark is reported in the text.

One confounder deserves separate treatment. Pilot cities start with higher pollution levels and end with lower levels than controls, which could also reflect convergence under the 2013 Air Pollution Prevention and Control Action Plan. Allowing a separate annual slope for 2016 baseline PM2.5 gives −3.21 μg/m^3^ (95% confidence interval −5.65 to −0.77); allowing baseline-tercile-specific year effects gives −3.70 (−5.89 to −1.50). A 2011–2012 baseline, preceding the national plan, yields −3.64 (−5.49 to −1.79). Table 4 presents these diagnostics alongside the main sensitivity results. They address these specified convergence paths rather than every possible differential trend.

Inference with 21 treated cities gives a wild-bootstrap *P*-value of 0.0067 and a 95% confidence set of [−6.75, −1.05] μg/m^3^ for the benchmark. Across 1,999 city-level reassignments, the coefficient-based permutation *P*-value is 0.010 and the studentised value is 0.013 (Appendix Figure A4). These are diagnostics under the stated reassignment scheme, given purposive historical assignment. Shifting the policy to 2014 within the pre-policy window gives +0.147 μg/m^3^ (95% interval −1.482 to +1.777; *P* = 0.859).

Precision is important alongside these results. Using the benchmark standard error and a cluster-t approximation, the effect associated with 80% power at a two-sided 5% level is approximately 3.74 μg/m^3^, close to the 3.87 estimate. This is an illustrative precision diagnostic based on the estimated standard error, not independent prospective verification of power. Smaller subgroups and interaction regressions may consequently leave substantively meaningful effects unresolved.

The modeled health translation also depends on the concentration–response assumptions. Table 5 reports the central GEMM, lower and upper risk-parameter endpoints, and the published fit excluding the Chinese male cohort (29). The estimated relative burden changes range from approximately −7.87% to −8.53% across these scenarios. Additional floor stress tests and the full parameter grid are in Appendix Table A2. These scenario-specific estimates and regression intervals do not validate absolute death counts or provide a joint interval for all health-impact uncertainty.

**Table 5:** Sensitivity of the modeled health translation.

Concentration–response scenario	Log coefficient	Change %	Conditional 95% CI %	*N*
GEMM θ lower endpoint	−0.0870	−8.33	−11.61, −4.92	3,135
Central GEMM	−0.0844	−8.10	−11.32, −4.76	3,135
GEMM θ upper endpoint	−0.0820	−7.87	−11.04, −4.59	3,135
GEMM excluding Chinese cohort	−0.0892	−8.53	−12.10, −4.83	3,135

Outcome: log one plus modeled attributable mortality. θ endpoints are 0.1430 ± 1.96 × 0.01807. Other central GEMM parameters remain fixed; the excluding-China scenario uses its own published parameter vector. Percentage changes equal 100[exp(β)−1] and approximately describe M when M is large. Intervals cover city-clustered regression uncertainty conditional on the selected scenario; they are not joint uncertainty intervals for the health-impact model. Every row uses the same controls and city/year effects.

### 4.6 Boundary conditions: moderation by measured conversion capacity

H2 predicts a stronger negative pilot–modeled-burden association where conversion capacity is greater. Table 6 reports all six corresponding interactions for modeled burden and PM2.5. None passes the 5% wild-bootstrap test. Smart-city status has a PM2.5 interaction of −4.69 μg/m^3^ (pointwise 95% interval −9.26 to −0.12), but its wild-bootstrap *P*-value is 0.066. Fiscal intensity has a modeled-burden interaction of −0.0442 (−0.0858 to −0.0026), with a wild *P* = 0.145. These estimates do not provide consistent support for H2; the intervals still allow for moderation of meaningful magnitude and do not establish equivalence.

**Table 6:** Baseline conversion capacity and the exposure-related burden.

Outcome	Baseline proxy	Interaction	95% CI	Wild *P*	Holm *P*
PM2.5	Telecom	−0.38	−3.36, 2.60	0.813	1.000
PM2.5	Smart	−4.69	−9.26, −0.12	0.066	0.395
PM2.5	Fiscal	−0.55	−4.93, 3.84	0.845	1.000
Log modeled burden	Telecom	−0.0070	−0.0537, 0.0397	0.792	1.000
Log modeled burden	Smart	0.0178	−0.0645, 0.1002	0.696	1.000
Log modeled burden	Fiscal	−0.0442	−0.0858, −0.0026	0.145	0.723

Each row includes the pilot term, its interaction, full controls, city/year effects, and baseline-carrier-by-year terms. Continuous 2016 carriers are standardized across cities; smart-city status is binary. *N* = 3,130 in each row. Intervals are pointwise 95% city-clustered intervals. Holm correction is applied to the six wild-bootstrap *P*-values in this table as one family, separately from the nine direct-capacity tests in Appendix Table 5A.

Appendix Tables A4, A5 compare specifications using baseline proxies with common year effects and contemporaneous proxies. The latter may respond to the programme and therefore mix effect modification with policy-induced changes in the moderator. These comparisons are exploratory and do not replace the baseline-proxy-by-year design or establish a particular institutional constraint.

The capacity indicators cannot distinguish the institutional explanations in Section 2.3. Physician training and bed construction may adjust slowly, while warnings, emergency services, prevention, and household avoidance may respond more rapidly. Employee-insurance enrolment does not capture these services and has only one pre-policy observation per city. Consequently, an imprecise insurance coefficient cannot rule out an adjustment-lag explanation or establish budget separation as the operative mechanism.

Table 7 examines conversion-capacity interactions directly involving physicians, beds, and employee-insurance enrolment as exploratory extensions. The pattern is mixed. The telecommunications–bed interaction is +4.40 beds per 10,000 residents per one-standard-deviation difference in baseline telecommunications intensity (95% pointwise interval +2.47 to +6.32; wild *P* = 0.0023; Holm-adjusted *P* = 0.0207 across nine tests). The other eight interactions do not pass this family adjustment. The bed interaction remains positive in the comparisons reported in Appendix Table A6, but its pre-policy event interactions yield *P* = 0.0455 conventionally and 0.1711 under the wild bootstrap. Alongside the bed-stock pre-trend concern, this prevents a clean causal interpretation.

**Table 7:** Pre-policy conversion capacity and directly measured defensive outcomes.

Outcome	2016 proxy	Interaction	95% CI	Wild *P*	Holm *P*
Physicians	Telecommunications	−0.12	−3.18, 2.94	0.982	1.000
Physicians	Smart-city status	−0.52	−4.08, 3.04	0.796	1.000
Physicians	Fiscal intensity	−0.35	−2.11, 1.41	0.702	1.000
Beds	Telecommunications	4.40	2.47, 6.32	0.002	0.021
Beds	Smart-city status	−2.96	−8.68, 2.75	0.340	1.000
Beds	Fiscal intensity	−2.58	−4.94, −0.22	0.052	0.419
Insurance ratio	Telecommunications	0.0117	−0.0056, 0.0290	0.183	1.000
Insurance ratio	Smart-city status	−0.0098	−0.0382, 0.0186	0.514	1.000
Insurance ratio	Fiscal intensity	−0.0004	−0.0181, 0.0173	0.969	1.000

Every row is a separate regression, including treatment, its proxy interaction, full controls, city/year effects, and proxy-by-year interactions. Continuous proxies are standardized across cities; smart-city status is binary. Physician and bed units are per 10,000 residents; insurance is a ratio. *N* = 3,130 for physician/bed rows and 1,827 for insurance. Intervals are pointwise city-clustered intervals, not simultaneous intervals. Holm correction is applied to the nine wild-bootstrap P-values (9,999 draws) as one family. The bed–telecommunications result is exploratory because pre-trend diagnostics differ by inference method.

The measured proxies, therefore, leave the role of conversion capacity unresolved. Telecommunications revenue, designation as a smart-city pilot, and fiscal expenditure intensity do not reflect the quality or effectiveness of implemented digital governance. The results cannot establish that municipal capability is irrelevant, that digital tools cannot improve protection, or that the programme's institutional design uniquely explains the observed pattern.

### 4.7 Heterogeneity: exploratory patterns by baseline conditions

Table 8 splits the sample by fixed 2016 terciles of PM2.5 and physician density. Formal pooled tests allow the control coefficients and year effects to vary across terciles, matching the separate subgroup regressions. They do not reject equal coefficients for baseline pollution (*P* = 0.4605) or physicians (*P* = 0.1774). Significance in one group and not another is not a test of their difference.

**Table 8:** PM2.5 heterogeneity by baseline exposure and physician density.

Split	Tercile	Pilots	Estimate	95% CI	Wild *P*	*N*
PM2.5	1	7	−2.72	−5.81, 0.38	0.141	1,044
PM2.5	2	6	−4.56	−7.23, −1.89	0.020	1,042
PM2.5	3	8	−1.70	−6.05, 2.65	0.463	1,044
Physicians	1	3	−5.67	−9.82, −1.51	0.090	1,054
Physicians	2	7	−0.69	−4.29, 2.90	0.723	1,044
Physicians	3	11	−4.01	−8.30, 0.28	0.098	1,032

All regressions include full controls and city/year effects. Terciles use fixed 2016 values. Intervals are pointwise 95% city-clustered intervals; wild P-values use 9,999 draws and are exploratory and unadjusted. Formal between-group tests fully interact the controls and year effects. The three-treated-city group's much wider wild confidence set is reported in the text.

Appendix Figure A3 displays the PM2.5 estimates. Across physician-density terciles, they are −5.67 μg/m^3^ in the lowest group, −0.69 in the middle, and −4.01 in the highest. These comparisons describe heterogeneity in the exposure outcome and cannot identify how physicians alter the mortality consequences of pollution. The modeled burden provides no independent evidence on that relationship.

The lowest physician-density tercile contains only three pilot cities. Its wild-bootstrap *P*-value is 0.0902, and the inverted 95% confidence set is extremely wide, approximately [−16.21, +99.39] μg/m^3^. The baseline-pollution split also does not follow a monotonic pattern. These estimates are exploratory, and neither precise subgroup effects nor distributional or equity conclusions follow from them.

## 5 Discussion

### 5.1 Theoretical implications

The contribution is to distinguish the exposure-related health gain from a possible additional defensive contribution. The co-benefits literature converts environmental estimates into health benefits through concentration–response functions; reduced exposure and its associated modeled health gain already constitute a co-benefit, without requiring an increase in health-system capacity. Naming the degree of coupling makes the additional defensive contribution explicit. Our physician-based calibration gives a ratio of 0.0062 (95% Fieller interval −0.0324 to +0.0462) at an assumed physician–mortality elasticity of −0.047. This conditional interval admits positive contributions and covers only one measured component of defense. It neither establishes H1b nor bounds the contribution of defensive adaptation as a whole.

That measurement point generalizes beyond this application. Wherever a health burden is computed from exposure through a fixed concentration–response function, the resulting variable is a deterministic transform of exposure and the accompanying demographic assumptions. It cannot register a defensive response that changes health at a given exposure while those assumptions remain fixed. Differences between estimated exposure and constructed-burden effects may instead reflect non-linear transformation or population weights. Evidence on defensive adaptation therefore requires independently measured capacity, service, or health outcomes. We regard this as the study's main methodological caution for a literature that increasingly relies on modeled burden measures.

The second theoretical implication concerns the distinction between a plausible institutional mechanism and evidence identifying it. The conversion-capacity intervals are too wide to establish equivalence, while the positive telecommunications–bed interaction is an exploratory association subject to pre-trend concerns. These results do not demonstrate a structural ceiling or locate a binding constraint in assessment design rather than municipal capability. The multitask argument offers a possible account of effort allocation, but testing it requires direct evidence on local evaluation rules, budgets, and implementation.

### 5.2 Dialogue with the literature

The findings speak to the defensive-investment tradition (5, 6, 20) by examining municipal capacity alongside pollution exposure. They provide a clearer reduction in exposure than a precisely estimated average expansion of the observed health-system inputs. That difference does not imply that public defensive adaptation remained inert: warnings, emergency coordination, preventive care, and household avoidance could change without expanding physician or bed stocks. This distinction complements evidence that pollution and its regulation affect observed health and related outcomes (39–43).

For the Chinese environmental-policy literature, the results qualify a common inference. Evaluations of low-carbon and carbon-market pilots typically establish emission or intensity effects (44, 45); Zheng and Kahn (46) document the broader progress of urban environmental quality. Our estimates support the exposure step for an adaptation programme, while the GEMM calculation describes only the health burden associated with that exposure. The multitask reading is consistent with evidence that Chinese local officials allocate effort toward measured objectives (28, 47), that mandates can displace effort across jurisdictions (48), and that changing what is measured alters what is delivered (49). These studies motivate an institutional explanation; they do not establish that explanation for the pilot examined here.

For public health, the results inform the interpretation and scale of the dividend. Estimates of the health co-benefits of climate policy are frequently large (23, 25, 50). Here, the central GEMM calculation translates the exposure change into an approximately 8.1% lower modeled pollution-related burden (95% confidence interval −11.3% to −4.8%), conditional on the specified accounting assumptions. This is neither a count of deaths prevented nor an estimate of the programme's total health benefit. Differences from other co-benefit projections can reflect policy scope, exposure changes, populations, and modeling assumptions, and cannot be attributed to an inactive defensive margin based on these data.

### 5.3 Policy implications

The policy reading is specific to China's 2017 climate-adaptive city pilot. The measured digital inputs provide mixed evidence: the exposure and modeled-burden interactions do not pass wild-bootstrap tests, whereas baseline telecommunications intensity is positively associated with the bed response, subject to pre-trend concerns. Telecommunications revenue and formal smart-city designation do not measure the quality of implemented digital governance. Investment in monitoring platforms, sensing networks, and artificial-intelligence-enabled surveillance may be valuable for many reasons, and these estimates cannot rule out additional health benefits from their effective use. They do not establish whether digital infrastructure is, or is not, the binding constraint.

Two evaluation priorities follow from these measurement limits. First, assess changes in exposure alongside observed health outcomes and the delivery of protective services. Second, examine whether coordination and resource allocation support those services. The national pilot documents explicitly include public health, warnings, emergency coordination, and financial support (12, 13). They therefore do not support a claim that the programme excluded health objectives or prohibited resources from supporting them. Local assessment rules, expenditure records, and implementation reports would be needed to test whether particular incentives or administrative arrangements constrained the response. These are questions for further institutional evaluation rather than mechanisms identified by the capacity estimates.

The scope is the 2017 pilot and the cities observed through 2022. Other adaptation programmes may fund different activities, place different weight on health services, or operate over a longer horizon. They could generate a positive defensive contribution even when the present indicators do not establish one. The evidence here should therefore not be generalized to all climate adaptation policies.

### 5.4 Scope conditions and limitations

Six limitations bound the claims. First, and most importantly, the health outcome is a constructed accounting quantity, not observed mortality; it inherits the assumptions of the GEMM function and of the national baseline rate and age share. It should be read as exposure translated into health units, not as counted deaths. The city-level calculation is ecological: annual averages do not capture individual differences in residential or workplace exposure, avoidance behavior, pre-existing disease, or actual mortality. The parameter scenarios examine selected assumptions rather than all uncertainties in health impact.

Second, with 21 treated clusters, statistical precision remains limited. Using the benchmark PM2.5 standard error, an illustrative cluster-t calculation gives an 80% power detection threshold of about 3.74 μg/m^3^, close to the estimated 3.87 μg/m^3^; this is not a prospective power assessment. The H2 results reflect an absence of precise evidence rather than equivalence findings. The smaller matched sample produces a non-significant estimate, and the physician-density subgroup containing only three treated cities supports no causal or equity conclusions.

Third, identification relies on conditional parallel trends and the absence of relevant differential shocks or spillovers. The single 2017 cohort does not separate the pilot from every contemporaneous change specific to treated cities, despite the pollution-convergence and concurrent-policy checks. Bed-stock pre-trends are jointly significant under conventional and wild-bootstrap inference; the telecommunications–bed pre-trend tests also raise concerns. These diagnostics restrict the causal interpretation of the capacity results. Broader city-level coding of some district or cross-city pilots is another source of uncertainty.

Fourth, the physician-based coupling calibration depends on transferring a United States primary-care elasticity (33) to Chinese city physician stocks. Differences in physician definitions, health systems, population needs, and access make that transfer uncertain. The wider elasticity grid shows assumption sensitivity: at an elasticity of −0.50, the ratio is 0.0663 with a 95% Fieller interval of −0.3449 to +0.4912. These intervals propagate joint regression-coefficient uncertainty conditional on the assumed elasticity and normalization, not uncertainty in the elasticity, the GEMM model, or unmeasured defense. They are illustrative calibrations, not bounds on total defensive adaptation.

Fifth, the digital proxies measure telecommunications activity and formal smart-city designation rather than the quality of digital governance. Fiscal expenditure intensity likewise does not identify spending directed toward protective services. Their interaction estimates cannot establish the effectiveness or irrelevance of well-implemented digital governance or targeted finance.

Sixth, the defensive indicators describe administrative stocks and enrolment rather than short-run service flows. They do not capture emergency response, heat-warning systems, public-health messaging, preventive-care activity, or household avoidance. A response confined to those activities would not appear in our data, and employee-insurance enrolment has only one pre-policy year. Future work could link warning issuance and reach, emergency-response records, preventive-service use, and household exposure information to local programme implementation and observed health outcomes. Such data would help test mechanisms that the present city-year stock measures leave unobserved.

## 6 Conclusion

Exposure reduction and defensive adaptation are distinct ways in which climate policy may benefit health. This study formalized their relationship as a coupling degree and examined the two margins in China's 2017 climate-adaptive city pilot. The distinction clarifies both the information supplied by exposure-based health accounting and the separate evidence needed to assess protective capacity.

The pilot is associated with a PM2.5 reduction of 3.87 μg/m^3^ (95% confidence interval −6.49 to −1.25). The negative association persists with concurrent-policy controls and baseline-pollution-specific trends, and remains significant under few-cluster inference, although the matched estimate is smaller and not statistically significant. The central GEMM calculation translates the exposure change into approximately 8.1% lower modeled pollution-related health burden (95% confidence interval −11.3% to −4.8%); it does not measure observed mortality. Average changes in physicians, beds, and employee-insurance enrolment remain imprecisely estimated. The positive but exploratory telecommunications–bed association, the wide moderation intervals, and the bed-related pre-trend concerns prevent a conclusion that the defensive contribution is absent or non-positive.

The practical conclusion is that air quality improvement is one relevant component of the health return to adaptation policy, while a broader evaluation requires evidence on protection and observed health. This programme is associated with cleaner air and a lower exposure-based modeled burden, but the available inputs do not reveal whether warnings, services, or other defensive responses have improved. Extending the evaluation to those activities, as well as to the local assessment and funding arrangements that support them, would help distinguish exposure-related co-benefits from additional gains through defensive adaptation.

## Data Availability

The original contributions presented in the study are included in the article/supplementary material, further inquiries can be directed to the corresponding authors.
