# Supplementary material for: Does climate adaptation policy deliver a health dividend? Exposure, modeled health burden, and defensive capacity in China's climate-adaptive city pilots
Source: Front Public Health. 2026 Oct 14;14:1987084. doi: 10.3389/fpubh.2026.1987084 (PMC13717398; doi:10.3389/fpubh.2026.1987084)
Supplement: Supplementary file 1 [file Supplementary_file_1.docx]

Supplementary Material

This appendix reports alternative estimators, accounting and calibration sensitivity, complete moderator comparisons, policy coding and the descriptive diagnostics referred to in the main text. PM2.5 and modelled burden are distinct outcomes, and estimation samples and weights differ across methods. The executable replication package contains the data dictionary, GEMM construction, estimation code and complete machine-readable outputs.

Table A1. Alternative estimators for PM2.5 and modelled attributable burden.

| **Estimator** | **Outcome** | **Estimate** | **95% CI** | ***p*** | **N** |
| --- | --- | --- | --- | --- | --- |
| Two-way fixed effects | PM2.5 | −3.87 | [−6.49, −1.25] | 0.004 | 3135 |
| Two-way fixed effects | Log modelled burden | −0.0844 | [−0.1201, −0.0487] | <0.001 | 3135 |
| Doubly robust | PM2.5 | −3.94 | [−6.32, −1.56] | 0.001 | 3130 |
| Doubly robust | Log modelled burden | −0.0799 | [−0.1257, −0.0340] | <0.001 | 3130 |
| Entropy balancing | PM2.5 | −2.98 | [−5.59, −0.37] | 0.026 | 3130 |
| Entropy balancing | Log modelled burden | −0.0822 | [−0.1168, −0.0476] | <0.001 | 3130 |
| Weighted matching | PM2.5 | −1.67 | [−4.92, 1.57] | 0.308 | 874 |
| Weighted matching | Log modelled burden | −0.0707 | [−0.1102, −0.0312] | <0.001 | 874 |

Note: City-clustered intervals are reported; doubly robust estimates use asymptotic normal inference, while fixed-effects estimates use residual cluster degrees of freedom. The common 2017 cohort has never-treated comparisons. Entropy balancing uses first moments of baseline covariates; matching uses three nearest neighbours and common support. The matched PM2.5 estimate is not significant at 5%. Health-accounting estimates remain model-dependent and do not constitute observed mortality evidence.

Figure A1. Construction of modelled attributable burden. The calculation maps PM2.5 through the GEMM hazard ratio and attributable fraction, then applies fixed mortality and adult-share assumptions and registered population. The concentration–response curve contains no defensive-capacity input and provides no observed death records.

Figure A2. Raw trends in pilot and comparison cities. Annual unweighted means of PM2.5 and modelled attributable rates in the benchmark sample. The vertical line marks the 2017 policy start. These descriptive trends do not adjust for covariates or identify the difference-in-differences association.

Figure A3. Exploratory PM2.5 heterogeneity. Separate regressions by fixed 2016 pollution or physician-density terciles, with pointwise 95% city-clustered intervals. Formal pooled tests allow subgroup-specific controls and year effects. Pilot-city counts are shown, and the three-pilot group requires the wide wild-bootstrap confidence set reported in Section 4.7.

Figure A4. City-reassignment diagnostic for PM2.5. Distribution of coefficients from 1,999 reassignments of 21 pilot cities with the policy date held fixed; the line marks the benchmark. The coefficient-based p value is 0.010 and the studentised value is 0.013. The exercise does not reproduce the historical purposive assignment mechanism.

Figure A5. Event study for modelled attributable burden. Outcome: ln(1 + modelled attributable deaths). Coefficients and pointwise 95% city-clustered intervals are relative to 2016; 2011–2012 are pooled. The pre-period joint p value is 0.503. The accounting series is derived from exposure and population and is not an independent mortality observation.

The parameter grid includes the central risk parameter θ = 0.1430 with standard error 0.01807, its ±1 and ±1.96 standard-error endpoints, and the published excluding-China fit (θ = 0.1231, α = 1.5, μ = 10.4, ν = 25.9). The central concentration floor is 2.4 μg/m³. Changing the floor to 0 or 5.9 is an illustrative stress test, not a new epidemiological fit. These estimates are conditional on fixed accounting inputs.

Table A2. Complete GEMM concentration–response scenarios.

| **Scenario** | **Log coefficient** | **Conditional 95% CI** | **Change %** | **N** |
| --- | --- | --- | --- | --- |
| θ − 1.96 SE | −0.0870 | [−0.1234, −0.0505] | −8.33 | 3135 |
| θ − 1 SE | −0.0857 | [−0.1218, −0.0496] | −8.21 | 3135 |
| Central GEMM | −0.0844 | [−0.1201, −0.0487] | −8.10 | 3135 |
| θ + 1 SE | −0.0832 | [−0.1185, −0.0479] | −7.98 | 3135 |
| θ + 1.96 SE | −0.0820 | [−0.1169, −0.0470] | −7.87 | 3135 |
| Floor = 0 stress test | −0.0761 | [−0.1073, −0.0450] | −7.33 | 3135 |
| Floor = 5.9 stress test | −0.1028 | [−0.1516, −0.0540] | −9.77 | 3135 |
| Excluding Chinese cohort | −0.0892 | [−0.1289, −0.0495] | −8.53 | 3135 |

Note: All models use full controls, city/year effects and city clustering, 2011–2022. θ scenarios use the original GEMM parameter uncertainty; the two floor changes are separate stress tests. Parameter endpoints are not joint health-impact confidence limits. Parameters follow Burnett et al. (2018) and the accompanying GEMM calculator.

The physician calibration scales the proportional physician-density change by each assumed elasticity and divides by the exposure-only log-rate coefficient. The conditional range holds the denominator fixed and scales the physician wild interval. The Fieller interval uses the joint city-clustered covariance of both regression coefficients. Neither covers external-parameter uncertainty or unobserved defensive contributions.

Table A3. Physician-based coupling under external elasticity scenarios.

| **Assumed elasticity** | **Ratio** | **Conditional range** | **95% Fieller interval** |
| --- | --- | --- | --- |
| 0.000 | 0.0000 | [−0.0000, 0.0000] | [−0.0000, 0.0000] |
| −0.010 | 0.0013 | [−0.0068, 0.0093] | [−0.0069, 0.0098] |
| −0.025 | 0.0033 | [−0.0169, 0.0233] | [−0.0172, 0.0246] |
| −0.047 | 0.0062 | [−0.0317, 0.0438] | [−0.0324, 0.0462] |
| −0.100 | 0.0133 | [−0.0675, 0.0931] | [−0.0690, 0.0982] |
| −0.200 | 0.0265 | [−0.1351, 0.1862] | [−0.1380, 0.1965] |
| −0.300 | 0.0398 | [−0.2026, 0.2794] | [−0.2070, 0.2947] |
| −0.500 | 0.0663 | [−0.3377, 0.4656] | [−0.3449, 0.4912] |

Note: Every row is conditional on the indicated elasticity and common physician mean. The zero-elasticity row is zero by assumption, not an empirical result. Positive and negative physician contributions are compatible with all non-zero scenarios. These intervals do not bound total defensive adaptation. No equivalence threshold is specified.

These specifications retain the fixed 2016 proxies but impose common year effects. All nine capacity interactions are shown so that the effect of allowing baseline-specific time paths can be assessed against Table 5A.

Table A4. Baseline capacity moderators with common year effects.

| **Outcome** | **Proxy** | **Interaction** | **95% CI** | ***p*** | **N** |
| --- | --- | --- | --- | --- | --- |
| Physicians | Telecom | 0.93 | [−1.91, 3.76] | 0.520 | 3130 |
| Physicians | Smart city | 1.04 | [−2.32, 4.40] | 0.544 | 3130 |
| Physicians | Fiscal | −1.03 | [−2.64, 0.58] | 0.209 | 3130 |
| Beds | Telecom | 5.74 | [3.89, 7.58] | <0.001 | 3130 |
| Beds | Smart city | −1.38 | [−6.84, 4.09] | 0.620 | 3130 |
| Beds | Fiscal | −2.41 | [−4.67, −0.15] | 0.037 | 3130 |
| Insurance ratio | Telecom | 0.0122 | [−0.0032, 0.0276] | 0.119 | 1827 |
| Insurance ratio | Smart city | −0.0064 | [−0.0321, 0.0193] | 0.623 | 1827 |
| Insurance ratio | Fiscal | −0.0059 | [−0.0221, 0.0102] | 0.469 | 1827 |

Note: City effects, common year effects and full controls are included. Conventional pointwise city-clustered p values are shown without multiplicity correction because these are specification comparisons. Table 5A provides the adjusted nine-test family for the flexible specification. Physicians and beds are measured per 10,000 residents; employee-insurance enrolment is a population ratio.

These comparisons use time-varying proxies in their original centred scales. Policy-induced moderator changes may confound interpretation. Their coefficients do not have the same units as the standardised baseline interactions.

Table A5. Contemporaneous capacity interactions.

| **Outcome** | **Proxy** | **Interaction** | **95% CI** | ***p*** | **N** |
| --- | --- | --- | --- | --- | --- |
| Physicians | Telecom | 0.24 | [−2.56, 3.04] | 0.867 | 3131 |
| Physicians | Smart city | 0.89 | [−2.38, 4.17] | 0.592 | 3135 |
| Physicians | Fiscal | −3.97 | [−17.04, 9.10] | 0.550 | 3135 |
| Beds | Telecom | 6.45 | [4.61, 8.29] | <0.001 | 3131 |
| Beds | Smart city | −1.43 | [−6.89, 4.03] | 0.607 | 3135 |
| Beds | Fiscal | −9.92 | [−31.20, 11.36] | 0.360 | 3135 |
| Insurance ratio | Telecom | 0.0051 | [−0.0033, 0.0136] | 0.233 | 1828 |
| Insurance ratio | Smart city | −0.0064 | [−0.0321, 0.0192] | 0.623 | 1832 |
| Insurance ratio | Fiscal | −0.0002 | [−0.1007, 0.1002] | 0.996 | 1832 |

Note: City/year effects, full controls and the time-varying proxy main effect are included. For the fiscal interaction, fiscal intensity enters the model once as a main effect. Intervals and p values are pointwise city-clustered and unadjusted. Physicians and beds are measured per 10,000 residents; employee-insurance enrolment is a population ratio.

The telecommunications–bed interaction remains positive across the specifications in Table A6. They do not resolve the average bed-stock pre-trend rejection or the differing conventional and wild pre-trend diagnostics for the interaction.

Table A6. Bed–telecommunications specification comparisons.

| **Specification** | **Interaction** | **95% CI** | **Wild *p*** | **N** |
| --- | --- | --- | --- | --- |
| Climate controls only | 3.94 | [1.94, 5.94] | 0.005 | 3130 |
| 2011–2012 telecom baseline | 4.39 | [2.15, 6.62] | 0.001 | 3120 |
| Exclude partial-area mappings | 4.49 | [2.11, 6.88] | 0.012 | 3082 |

Note: The interaction remains positive in these comparisons, but this does not resolve the pre-trend concern. Models include city/year and baseline-proxy-by-year effects; other details follow the row labels. Wild-bootstrap tests use 9,999 draws.

The 21 panel treatment codes below are not a one-to-one representation of all 28 localities in the national notification. The four broader-area mappings motivate the exclusion check in Table 4; all retained panel cities outside the list form the comparison group.

Table A7. Treated panel units and policy geography.

| **City code** | **Panel city** | **Policy correspondence** |
| --- | --- | --- |
| 150100 | Hohhot | City named in the policy |
| 210200 | Dalian | City named in the policy |
| 211300 | Chaoyang | City named in the policy |
| 331100 | Lishui | City named in the policy |
| 340100 | Hefei | City named in the policy |
| 340600 | Huaibei | City named in the policy |
| 360400 | Jiujiang | City named in the policy |
| 370100 | Jinan | City named in the policy |
| 410500 | Anyang | City named in the policy |
| 420100 | Wuhan | City named in the policy |
| 420300 | Shiyan | City named in the policy |
| 430600 | Yueyang | City named in the policy |
| 430700 | Changde | City named in the policy |
| 460100 | Haikou | City named in the policy |
| 500000 | Chongqing | Bishan and Tongnan districts |
| 520200 | Liupanshui | City named in the policy |
| 610100 | Xi’an | Xixian New Area proxy |
| 610400 | Xianyang | Xixian New Area proxy |
| 611000 | Shangluo | City named in the policy |
| 620400 | Baiyin | City named in the policy |
| 630100 | Xining | Huangzhong County |

Note: Source: the 2017 official notification and the panel treatment coding. Xi’an and Xianyang are both proxies for the cross-city Xixian New Area; they should not be read as two independent whole-city designations. These four broader-area units are excluded together in the spatial-coding sensitivity.
